# Neuroprotective Effects of RNS60 in TDP‐43 Pathology‐Associated Amyotrophic Lateral Sclerosis

**DOI:** 10.1002/mus.70289

**Published:** 2026-06-04

**Authors:** Danny R. Vesevick, Supurna Ghosh, Andreas Kalmes, P. Hande Ozdinler, Mukesh Gautam

**Affiliations:** ^1^ Davee Department of Neurology and Clinical Neurological Sciences Northwestern University, Feinberg School of Medicine Chicago Illinois USA; ^2^ Revalesio Tacoma Washington USA; ^3^ Les Turner ALS Center Northwestern University, Feinberg School of Medicine Chicago Illinois USA; ^4^ Chemistry of Life Processes Institute Northwestern University Evanston Illinois USA; ^5^ Robert H. Lurie Comprehensive Cancer Center Northwestern University, Feinberg School of Medicine Chicago Illinois USA; ^6^ Mesulam Center for Cognitive Neurology and Alzheimer's Disease Northwestern University Chicago Illinois USA

## Abstract

**Introduction:**

TDP‐43 pathology is broadly observed in the cerebral cortex of patients with amyotrophic lateral sclerosis (ALS). RNS60, an experimental treatment for acute ischemic stroke and ALS, enhanced mitochondrial biogenesis and function in other preclinical models. We investigated whether RNS60 improved mitochondrial stability and upper motor neuron (UMN) health in a TDP‐43 mouse model of ALS.

**Methods:**

prpTDP‐43^A315T^‐UeGFP mice, in which UMNs express green fluorescent protein (eGFP), and WT‐UeGFP mice were treated with RNS60 or placebo intraperitoneally every other day from post‐natal day (P) 30 until P90. Astrogliosis and microgliosis in brain and spinal cord were quantified by immunocytochemistry. Mitochondrial ultrastructure was studied via electron microscopy, and mitochondrial function was assessed using flow cytometry. Neuromuscular junction (NMJ) integrity was assessed in gastrocnemius, tibialis, and diaphragm muscles.

**Results:**

RNS60 treatment reduced defective mitochondria in UMNs (prpTDP‐43^A315T^ + vehicle: 53.2% ± 0.71%; prpTDP‐43^A315T^ + RNS60: 19.6% ± 1.4%, *p* = 0.0001) and spinal motor neurons (prpTDP‐43^A315T^ + vehicle: 70.1% ± 0.4.48%; prpTDP‐43^A315T^ + RNS60: 33.5% ± 4.43%, *p* = 0.001). It increased mitochondrial membrane polarization (prpTDP‐43^A315T^‐UeGFP + vehicle: 7184 ± 1689 mean intensity; prpTDP‐43^A315T^‐UeGFP+RNS60: 22120 ± 4818 mean intensity, *p* = 0.032), reduced the extent of astrogliosis and microgliosis in motor cortex and spinal cord, protected UMNs compared to placebo, and enhanced the proportion of intact NMJs in leg and diaphragm muscles (prpTDP‐43^A315T^‐UeGFP + vehicle: 29.6% ± 3.6%; prpTDP‐43^A315T^‐UeGFP + RNS60: 64.3% ± 4.4%, *p* = 0.0002).

**Discussion:**

These results suggest that RNS60 treatment promotes motor neuron health in ALS by protecting mitochondrial structure and function, preserving NMJ integrity, and reducing gliosis.

**Abbreviations**
ALS: Amyotrophic Lateral Sclerosis
DNA: Deoxyribonucleic Acid
GFA: Glial Fibrillary Acidic Protein
Iba1: Ionized Calcium‐Binding Adapter Molecule 1
NMJ: Neuromuscular Junction
P30: Postnatal Day 30
P90: Postnatal Day 90
PFA: Paraformaldehyde
SMN: Spinal Motor Neuron
SOD1: Superoxide Dismutase 1
TDP‐43: TAR DNA‐binding Protein 43
TMRE: Tetramethylrhodamine Ethyl Ester
UCHL1: Ubiquitin C‐terminal hydrolase L1
UMN: Upper Motor Neuron

## 1 Introduction

Intracellular aggregation of TDP‐43 is detected in the brains of approximately 97% of sporadic and familial amyotrophic lateral sclerosis (ALS) patients [1, 2, 3]. An important outcome of TDP‐43 dysregulation is mitochondrial dysfunction [4, 5, 6, 7, 8, 9]. Previous studies in the prpTDP‐43^A315T^ mouse model revealed that the cellular pathologies and problems observed in the UMNs are almost identical to those in the UMNs of ALS patients with TDP‐43 pathology, including extensive gliosis and mitochondrial defects [10, 11]. This makes the model particularly suitable to test candidate drugs like RNS60. Therefore, we crossed prpTDP‐43^A315T^ mice with UCHL1‐eGFP mice [12] to enable cell‐type specific investigation of UMNs, which are important for the initiation and modulation of voluntary movement and which display early vulnerability in ALS [12, 13, 14, 15].

RNS60 is an experimental drug under clinical testing for acute ischemic stroke and ALS [16]. In a Phase 2 trial in ALS, RNS60 treatment for 24 weeks resulted in a slowing of respiratory function decline, measured by forced vital capacity (FVC), and an improvement of median survival time compared to placebo treatment [17, 18].

RNS60 has shown anti‐inflammatory and neuroprotective effects in a multitude of rodent models of chronic neurodegenerative diseases as well as acute neuronal injuries including acute ischemic stroke [19, 20, 21, 22, 23, 24, 25, 26]. It has also been reported to activate mitochondrial biogenesis in cultured neurons [20] and to promote differentiation and survival of oligodendrocytes, which was associated with higher spare respiratory capacity, indicating increased mitochondrial energy metabolism [24].

This study was conducted to investigate the therapeutic potential of RNS60 treatment against TDP‐43 pathology and to specifically study any effects on the affected neuronal mitochondria in the prpTDP‐43^A315T^ mouse model of ALS.

## 2 Methods

### 2.1 Animals

All animal procedures were approved by the Northwestern University Animal Care and Use Committee (IACUC) and complied with the standards of the National Institutes of Health (NIH). All mice were in a C57BL/6J background. UCHL1‐eGFP mice were generated in the Ozdinler Laboratory and are now available at the Jackson Laboratory (022476). Hemizygous UCHL1‐eGFP females mated with hemizygous prpTDP‐43^A315T^ males [27] (Jackson Laboratory, #: 010700) to generate prpTDP‐43^A315T^‐UeGFP mice. prp‐hTDP‐43^A315T^ mice were fed with gel diet (DietGel76A, ClearH_2_O, ME) to eliminate gastrointestinal problems. In prpTDP‐43^A315T^ mice, the human TDP‐43 gene with an *A315T* point mutation and a FLAG‐tag has been expressed under the prion promoter. FLAG‐tag is a short polypeptide protein tag (sequence *DYKDDDDK*) used to detect expression of human TDP‐43A315T. Transgenic mice were identified as described [10, 28].

### 2.2 In Vivo Administration of RNS60

RNS60 was provided by Revalesio (Tacoma, WA). RNS60 is oxygen supersaturated in 0.9% sodium chloride via proprietary technology that involves modified Taylor‐Couette‐Poiseuille flow. RNS60 has a dissolved oxygen concentration between 40 mg/L and 60 mg/L. prpTDP‐43^A315T^‐UeGFP and WT‐UeGFP mice were randomly segregated into different treatment groups. Mice received 300 μL RNS60 or 0.9% saline vehicle (negative control) by intraperitoneal (IP) injection every other day from P30 through P90 (Figure S1).

### 2.3 Tissue Preparation and Immunocytochemistry

Mice were deeply anesthetized and perfused with PBS followed by 4% paraformaldehyde (PFA) after receiving the last dose at P90. The brain, spinal cord, diaphragm muscle, tibialis muscle, and gastrocnemius muscles were dissected out followed by post‐fixation in 4% PFA overnight. Brain and spinal cord were sectioned at 50 μm using a Leica vibratome (Leica VT1000S, Leica Inc., Germany). Serial floating sections (300 μm apart) were processed for GFP, GFAP, and Iba1 immunohistochemistry as described [10, 11]. Muscle tissue was embedded in cryo‐OCT medium, and 30‐μm sections were prepared using a cryostat (Leica). Cryostat sections were stored at −20°C until being used for immunostaining.

The following antibodies are used: anti‐GFP (1:1000, Invitrogen), anti‐FLAG clone M2 (1:500, Sigma), anti‐GFAP (1:1000; Invitrogen), and anti‐Iba1 (1:500; Wako). Appropriate secondary fluorescent antibodies (1:500, AlexaFluor‐conjugated, Invitrogen) were added to the blocking solution at room temperature for 2 h in the dark. Nuclei were counterstained with 4′,6‐diamidino‐2‐phenylindole (DAPI). For staining of neuromuscular junctions, anti‐UCHL1 antibody (1:500, Proteintech) and α‐bungarotoxin conjugated with Alexa Fluor 555 (1:500, Invitrogen) were used.

### 2.4 Immuno‐Coupled Electron Microscopy

Coronal sections of brain spanning motor cortex and lumbar spinal cord sections were fixed with 4% PFA and 2% glutaraldehyde for 2 h at room temperature. Immunostaining was performed using Ctip2 (1:500, Sigma) antibody and developed with DAB immunostaining (Vector Sciences) to label UMNs in motor cortex. Spinal cord sections were not subjected to immunostaining as SMN can be identified by their large size in the ventral horn. Sections were washed with 0.12 M phosphate buffer pH 7.4 and then treated with 1% osmium tetroxide in 0.12 M phosphate buffer pH 7.4 for 1 h at room temperature. Sections were washed three times with 0.12 M phosphate buffer followed by treatment with 10% UranyLess (Electron Microscopy Sciences) for 1 h at room temperature. Sections were dehydrated by an ascending series of alcohol concentrations (50%, 70%, 80%, 90%, and 100%) and propylene oxide (Electron Microscopy Sciences) followed by treatment with epoxy resin for 24 h on a shaker. Sections were treated with resin for 7 h at room temperature followed by flattening between transparency sheets and cured in an oven at 65°C for 3 days. Motor cortex from brain and ventral horn from spinal cord sections was carefully dissected out, mounted on resin blocks, and cured in an oven at 65°C for 3 days. The blocks were trimmed before proceeding for semi‐thin and ultra‐thin sectioning. Resin blocks were sectioned on a Leica Ultracut UC6 ultramicrotome (Leica Inc., Nussloch, Germany). Sections (70 nm) were collected on 200 mesh copper‐palladium grids. Ultra‐thin sections were counterstained on a drop of UranyLess solution (Electron Microscopy Sciences) and 0.2% lead citrate (Electron Microscopy Sciences). Grids were examined on FEI Tecnai Spirit G2 TEM (FEI Company, Hillsboro, OR), and digital images were captured on an FEI Eagle camera.

For EM data analysis, UMNs and SMNs from three independent experiments were used. For both UMN and SMN, approximately 10 neurons per mouse were imaged and used for quantification. The total number of mitochondria, number of mitochondrial clumps per UMN, and percentage of mitochondria with cristae were quantified. Statistical analysis was performed using one‐way ANOVA with GraphPad Prism software.

### 2.5 Mitochondria Polarity Assay

Motor cortices from WT‐UeGFP and prpTDP‐43^A315T^‐UeGFP mice treated with RNS60 or vehicle from P30 to P90 were dissected and digested with papain enzyme (100 U/mL, Worthington) and DNase enzyme (100 U/mL, NEB) solution at 37°C for 20 min. After digestion, the enzymatic reaction was stopped by adding Ovo‐BSA solution, washing with Opti‐MEM, and gentle trituration to prepare a single cell suspension, as described [29]. Single cell suspension was treated with 50 nM TMRE (Abcam) prepared in SFM for 20 min and incubated at 37°C. Cells were centrifuged at 80 rcf for 5 min at room temperature, and the cell pellet was resuspended in 500 μL SFM containing 50 nM TMRE. Thereafter, cells were subjected to flow cytometry analysis using FACSymphony A5 High‐Parameter SORP Cell Analyzer (BD Bioscience). To analyze the TMRE accumulation in GFP^+^ neurons, first all events were gated to exclude debris and small cells. This population of cells was then gated to include live cells and exclude DAPI positive dead cells. Further gate was set up to select GFP^+^ cells. The intensity of red TMRE signal was measured in eGFP^+^ neurons, and the percentage of TMRE accumulation was calculated in eGFP^+^ neurons for each experiment.

### 2.6 Imaging and Data Collection for in Vivo Study

A Nikon AXR confocal microscope (Nikon Inc., Melville, NY) was used to acquire low‐ and high‐magnification images. EM grids were examined on FEI Tecnai Spirit G2 TEM (FEI Company) and digital images were captured on a FEI Eagle camera. EM imaging was performed at the Center for Advanced Microscopy/Nikon Imaging Center (CAM), Northwestern University Feinberg School of Medicine, Chicago.

### 2.7 Quantification of UMNs, Astrocytes, and Microglia

UMNs, astrocytes, and microglia were quantitatively assessed by counting numbers of GFP^+^ UMNs, GFAP^+^ astrocytes, and Iba1^+^ microglia in a defined area of motor cortex. Three matching sections that contained motor cortex were selected from each mouse, and the area of M1 motor cortex was defined as per Paxinos mouse brain atlas. Three well‐defined sections spanning the motor cortex (Section 1: Bregma 1.18 mm, interaural 4.98 mm; Section 2: Bregma 0.86 mm; interaural 4.66 mm; Section 3: Bregma 0.38 mm, interaural 4.18 mm) were used per mouse. Images were taken with a 10× objective on an epifluorescent microscope (Nikon).

GFP^+^ SMN, GFAP^+^ astrocytes, and Iba1^+^ microglia were assessed in three sections (600 μm apart) in the ventral horn of the lumbar spinal cord. Images were taken with a 10× objective on an epifluorescent microscope (Nikon).

### 2.8 Quantification of Mitochondria Ultrastructure

Quantification of mitochondrial ultrastructure was performed using high magnification electron microscopy images. First, the total number of mitochondria in a neuron was counted (1900×). Thereafter, mitochondrial ultrastructure was visualized at higher magnification (11,000× for cristae structure assessment). Mitochondria with visible cristae structures were counted as intact mitochondria. For the quantification of clumps of mitochondria, three or more mitochondria attached with each other were considered as one clump. All clumps in a neuron were counted. The above‐mentioned quantification strategy was applied to quantitatively assess mitochondrial ultrastructure for both UMN and SMN.

### 2.9 Quantification of Neuromuscular Junctions (NMJ)

30‐μm cryosections were prepared from gastrocnemius, tibialis, and diaphragm muscles. Images were captured at 10× using an epifluorescent microscope (Nikon). Sections from different depths of muscles were used for analysis. To quantify intact NMJ, we first counted α‐bungarotoxin stained motor end plate structures in an image in the red channel only. After that, the green channel was used to superimpose the nerve ending onto the motor end plate. NMJs with perfectly overlapping immunostaining for both UCHL1 (green) and α‐bungarotoxin (red) staining were considered intact NMJ. Total numbers of NMJ counted in gastrocnemius, tibialis, and diaphragm muscle are given in Table S1.

### 2.10 Statistical Analysis

All analyses were performed using GraphPad Prism (San Diego, CA, USA) software in a blinded fashion, whereby the individual performing the analyses was blind to the genotype and treatment groups. The D'Agostino and Pearson normality test was performed on all datasets. For comparison of two groups, an unpaired Student *t*‐test with Welch's correction was used. Statistical differences between more than two groups were determined by one‐way ANOVA followed by the Tukey's *post hoc* multiple‐comparison test. Statistically significant differences were taken at *p* = 0.05.

## 3 Results

### 3.1 RNS60 Treatment Reduces Gliosis

**FIGURE 1:**
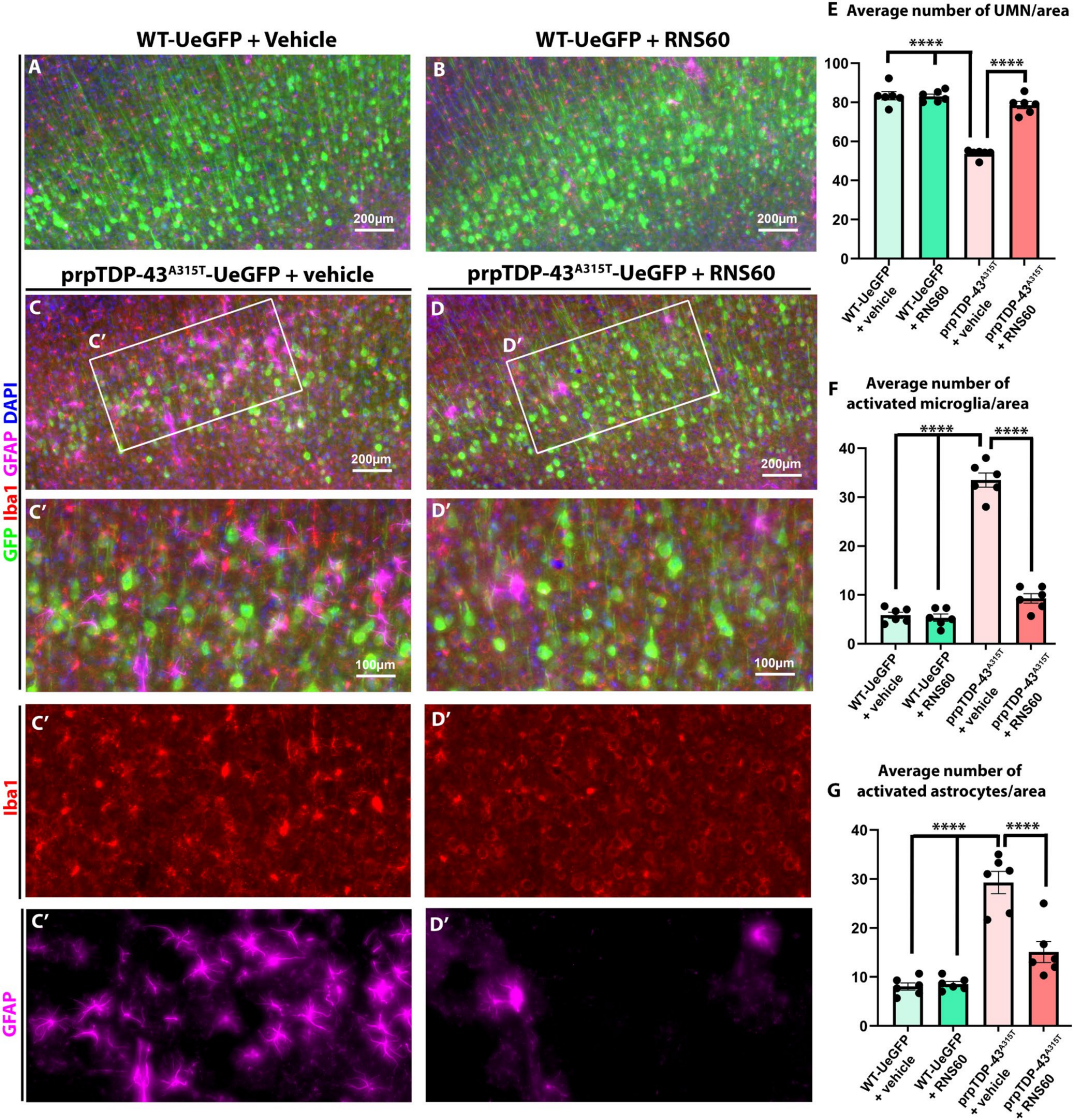
RNS60 provides neuroprotection and reduces gliosis in TDP‐43 ALS motor cortex. (A) A representative image of the motor cortex of WT‐UeGFP mouse treated with vehicle, (B) WT‐UeGFP mouse treated with RNS60, (C) a prpTDP‐43^A315T^‐UeGFP mouse treated with vehicle, and (D) prpTDP‐43^A315T^‐UeGFP mouse treated with RNS60. (C', D') Magnified images of inset. (E) Quantification of the average number of UMN/area. (F) Quantification of the average number of activated microglia/area. (G) Quantification of the average number of activated astrocytes/area. ****p = 0.0001. Scale bar: 200 μm (A–D), 100 μm (C'–D').

The average number of activated microglia in the motor cortex of prpTDP‐43^A315T^‐UeGFP mice treated with RNS60 was significantly lower than that of mice treated with vehicle (prpTDP‐43^A315T^‐UeGFP+RNS60: 9.28 ± 0.96, *n* = 6; prpTDP‐43^A315T^‐UeGFP+vehicle: 33.5 ± 1.43, *n* = 6; *p* = 0.0001; Figure 1C,C′,D,D′,F). On the other hand, the average numbers of microglia in healthy mice with or without RNS60 treatment were comparable (WT‐UeGFP +: 5.8 ± 0.6, *n* = 6; WT‐UeGFP+RNS60: 5.33 ± 0.73, *n* = 6; *p* = 0.983; Figure 1A,B,F).

Similarly, astrogliosis was reduced in the motor cortex upon RNS60 treatment, as shown by lower numbers of activated astrocytes (prpTDP‐43^A315T^‐UeGFP + vehicle: 29.9 ± 2.27, *n* = 6; prpTDP‐43^A315T^‐UeGFP + RNS60: 15.1 ± 2.15, *n* = 6; *p* = 0.0001 compared to prpTDP‐43^A315T^‐UeGFP + vehicle; Figure 1C,C′,D,D′,G). The average numbers of activated astrocytes were comparable in all WT mice regardless of treatment (WT‐UeGFP + vehicle: 8 ± 0.73, *n* = 6; WT‐UeGFP + RNS60: 8.5 ± 0.53, *n* = 6; *p* = 0.996; Figure 1A,B).

To assess whether reduction of gliosis is restricted to the motor cortex, we next investigated the spinal cord (Figure S2). Microglial numbers in WT mice were comparable regardless of treatment (WT‐UeGFP + vehicle: 13.8 ± 2.7, *n* = 6; WT‐UeGFP + RNS60: 15.5 ± 2.7, *n* = 6; Figure S2A,A',B,B',F). However, RNS60 treatment significantly reduced microgliosis in the spinal cord of prpTDP‐43^A315T^‐UeGFP mice (prpTDP‐43^A315T^‐UeGFP + vehicle: 40.3 ± 3.7*n* = 6; prpTDP‐43^A315T^‐UeGFP + RNS60: 22.9 ± 5.2, *n* = 6; *p =* 0.0004; Figure S2C,C′,D,D′,F) [30]. Likewise, the extent of astrogliosis was reduced in the spinal cord upon RNS60 treatment, while astrogliosis in WT animals remained comparable between RNS60 and vehicle treatment (WT‐UeGFP + vehicle: 30.7 ± 4.4, *n* = 6; WT‐UeGFP + RNS60: 25.6 ± 2.6, *n* = 6; Figure S2A,A',B,B′; prpTDP‐43^A315T^‐UeGFP + vehicle: 53.9 ± 7.1, *n* = 6; *p =* 0.0137; prpTDP‐43^A315T^‐UeGFP +RNS60: 26.7 ± 2.7, *n* = 6; *p =* 0.0024; Figure S2C,C′,D,D′,G).

### 3.2 RNS60 Treatment Preserves UMNs in the Motor Cortex

The average number of UMNs in the motor cortex of prpTDP‐43^A315T^‐UeGFP mice treated with RNS60 was significantly higher than that of prpTDP‐43^A315T^‐UeGFP mice treated with vehicle, and comparable to that in healthy WT mice (WT‐UeGFP + vehicle: 83.4 ± 2, *n* = 6; WT‐UeGFP + RNS60: 83 ± 1.16, *n* = 6; prpTDP‐43^A315T^‐UeGFP + vehicle: 53.7 ± 0.89, *n* = 6; *p* = 0.0001; prpTDP‐43^A315T^‐UeGFP + RNS60: 78.6 ± 1.87, *n* = 6; *p* = 0.0001; Figure 1A–D′,E).

Since the prpTDP‐43^A315T^ mouse model of ALS does not display a significant amount of spinal motor neuron loss, we did not find any differences in SMN numbers regardless of treatment, as expected (WT‐UeGFP + vehicle: 7.8 ± 1.07, *n* = 6; WT‐UeGFP + RNS60: 5.5 ± 0.51, *n* = 6; prpTDP‐43^A315T^‐UeGFP + vehicle: 5.15 ± 1.3, *n* = 6; *p =* 0.995; prpTDP‐43^A315T^‐UeGFP + RNS60: 6.18 ± 1.5, *n* = 6; *p* = 0.924; Figure S2A–D',E).

### 3.3 RNS60 Treatment Improves Mitochondrial Ultrastructure in Diseased UMNs

**FIGURE 2:**
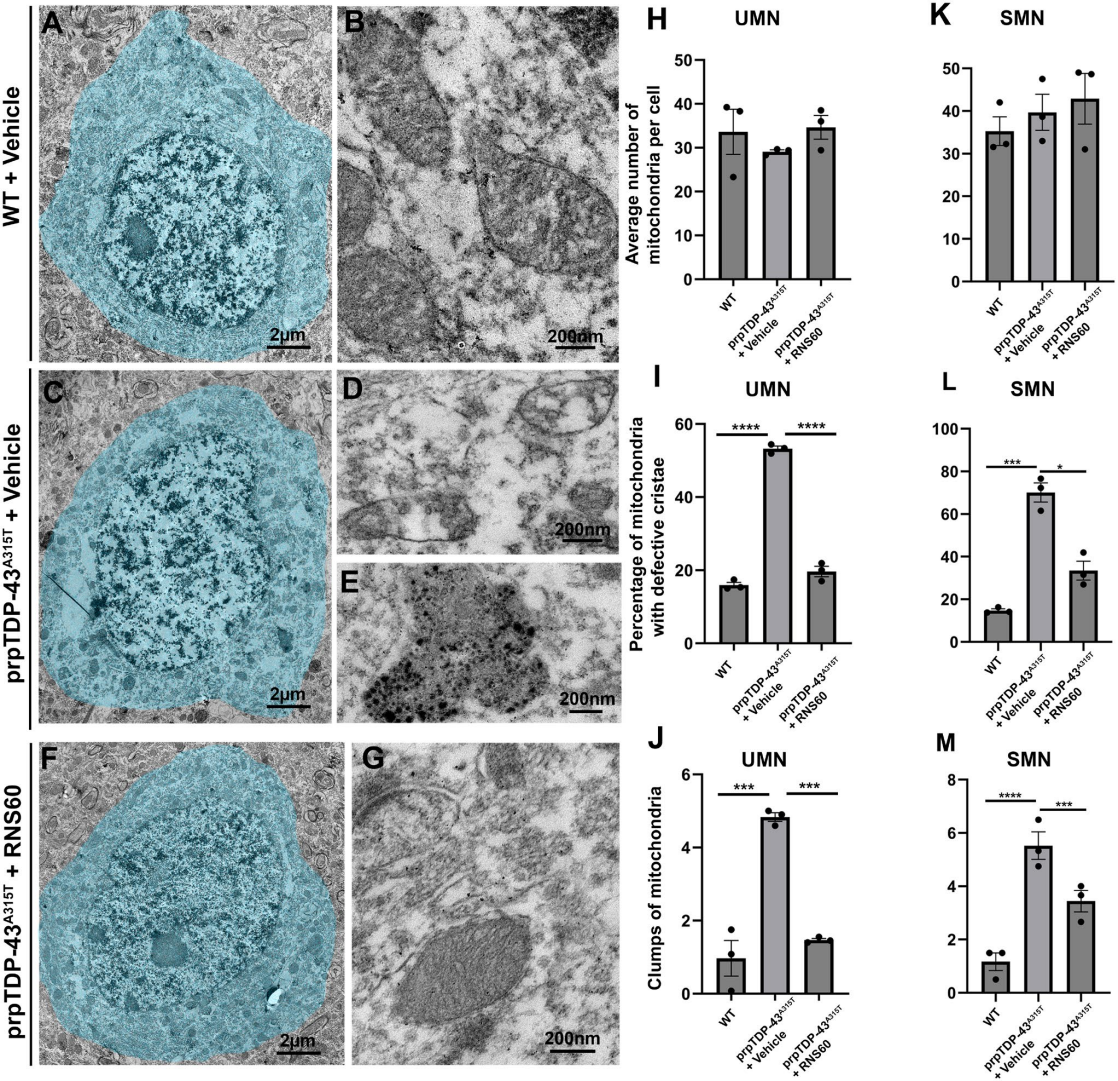
Treatment with RNS60 improves mitochondrial ultrastructure. (A) A representative electron microscopy image of Ctip2‐postive UMN from a WT mouse treated with vehicle. (B) Zoomed‐in representative images of mitochondria from Ctip2‐postive UMN from a WT mice treated with vehicle. (C) A representative electron microscopy image of Ctip2‐postive UMN from a prpTDP‐43^A315T^‐UeGFP mice treated with vehicle. (D, E) Zoomed‐in representative images of mitochondria from Ctip2‐postive UMN of prpTDP‐43^A315T^‐UeGFP mice treated with vehicle. (F) A representative electron microscopy image of Ctip2‐postive UMN from prpTDP‐43^A315T^‐UeGFP mice treated with RNS60. (G) Zoomed‐in representative images of mitochondria from Ctip2‐postive UMN of prpTDP‐43^A315T^‐UeGFP treated with RNS60. (H) Quantification of the average number of mitochondria per UMN, (I) average percentage of mitochondria with defective cristae per UMN, and (J) average number of mitochondria clumps per UMN. (K) Quantification of the average number of mitochondria per SMN, (L) average percentage of mitochondria with defective cristae per SMN, and (M) average number of mitochondria clumps per SMN. The perimeter of Ctip2 + UMN soma is marked with teal color (A, D, F). *p = 0.01, ***p = 0.004, ****p = 0.0001. Scale bar: 2 μm (Figure 2A,C,F), 200 nm (Figure 2B,D,E,G).

The average percentage of mitochondria with defective cristae was significantly reduced upon RNS60 treatment as compared to vehicle treated mice (WT‐UeGFP + vehicle: 15.94% ± 0.76%, *n* = 3; prpTDP‐43^A315T^‐UeGFP + vehicle: 53.2% ± 0.71%, *n* = 3; prpTDP‐43^A315T^‐UeGFP + RNS60: 19.6% ± 1.4%, *n* = 3; *p* = 0.0001; Figures 2A–G,I and S3A–K). In addition, mitochondrial aggregates in UMNs of prpTDP‐43^A315T^‐UeGFP mice were significantly reduced upon RNS60 treatment when compared to vehicle treatment (WT‐UeGFP + vehicle: 1 ± 0.48, *n* = 3; prpTDP‐43^A315T^‐UeGFP + vehicle: 4.83 ± 0.21, *n* = 3; *p =* 0.0002; prpTDP‐43^A315T^‐UeGFP + RNS60: 1.46 ± 0.04, *n* = 3; *p* = 0.0004; Figures 2A–G,J and S3A–K). However, the total number of mitochondria in UMNs of WT and prpTDP‐43^A315T^‐UeGFP mice were comparable regardless of treatment (WT‐UeGFP + vehicle: 33.6 ± 5.1, *n* = 3; prpTDP‐43^A315T^‐UeGFP + vehicle: 29.1 ± 0.41, *n* = 3; *p* = 0.633; prpTDP‐43^A315T^‐UeGFP + RNS60: 34.6 ± 2.7, *n* = 3; *p =* 0.514; Figure 2H).

Even though there is no aberrant SMN loss in prpTDP‐43^A315T^ mice, ultrastructural studies suggest the presence of impaired mitochondria [28]. We therefore investigated whether RNS60 treatment improves mitochondrial integrity in SMNs. The total numbers of SMN mitochondria were comparable across all genotypes and treatment groups (WT‐UeGFP + vehicle: 35.3 ± 3.3, *n* = 3; prpTDP‐43^A315T^‐UeGFP + vehicle: 39.7 ± 4.23, *n* = 3; *p =* 0.515; prpTDP‐43^A315T^‐UeGFP + RNS60: 42.9 ± 5.9, *n* = 3; *p =* 0.881; Figure 2K). However, the percentage of mitochondria with defective cristae structures were significantly higher in the SMN of prpTDP‐43^A315T^ mice, and RNS60 treatment significantly preserved the integrity of the cristae structures, making them comparable to WT healthy controls (WT‐UeGFP + vehicle: 14.7% ± 0.79%, *n* = 3; prpTDP‐43^A315T^‐UeGFP + vehicle: 70.1% ± 0.4.48%, *n* = 3; *p =* 0.0001; prpTDP‐43^A315T^‐UeGFP + RNS60: 33.5% ± 4.43%, *n* = 3; *p* = 0.001; Figures 2L and S4A–I). In addition, there were significantly more aggregates of defective mitochondria in SMN of prpTDP‐43^A315T^ mice compared to WT, and RNS60 treatment significantly improved the extent of these aggregates (WT‐UeGFP + vehicle: 1.17 ± 0.33, *n* = 3; prpTDP‐43^A315T^‐UeGFP + vehicle: 5.52 ± 0.51, *n* = 3; *p* = 0.008; prpTDP‐43^A315T^‐UeGFP + RNS60: 3.44 ± 0.4, *n* = 3; *p* = 0.03; Figures 2M and S4A–I).

### 3.4 RNS60 Improves Mitochondrial Function in Diseased UMNs

**FIGURE 3:**
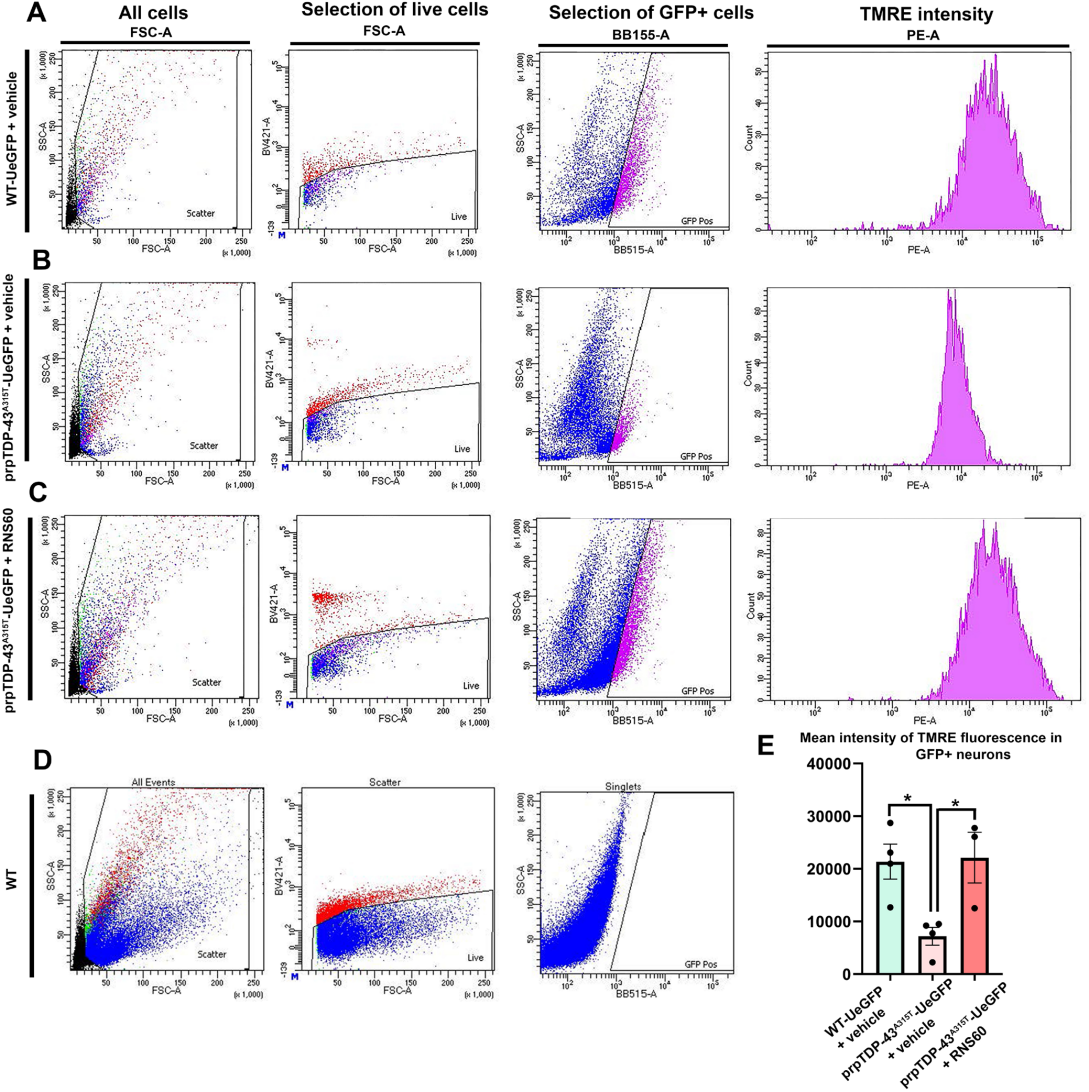
RNS60 enhances mitochondrial membrane polarization. (A) Representative gating strategy for flow cytometry analysis of WT‐UeGFP mice motor cortex single cell suspension treated with vehicle. (B) Representative gating strategy for flow cytometry analysis of prpTDP‐43^A315T^‐UeGFP mice motor cortex single cell suspension treated with vehicle. (C) Representative gating strategy for flow cytometry analysis of prpTDP‐43^A315T^‐UeGFP mice motor cortex single cell suspension treated with RNS60. (D) Representative gating strategy for flow cytometry analysis to show GFP negative cells in the WT mice. (E) Quantification of levels of TMRE in GFP‐positive UMN. *p = 0.032.

We investigated whether the preservation of mitochondrial membrane ultrastructure by RNS60 in UMNs translated into improved mitochondrial function by measuring the levels of tetramethylrhodamine ethyl ester (TMRE), a cationic dye that accumulates in mitochondrial membrane. Higher levels of TMRE correlate with higher mitochondrial membrane potential across the inner mitochondrial membrane, and thereby greater mitochondrial function. In addition to vehicle‐treated prpTDP‐43^A315T^‐UeGFP mice, WT‐UeGFP mice treated with vehicle were used as a second control. TMRE levels were significantly lower in UMNs of prpTDP‐43^A315T^‐UeGFP mice compared to that of WT mice, but significantly improved upon RNS60 treatment to similar levels as in WT mice, suggesting that RNS60 improves mitochondrial function by protecting mitochondrial ultrastructure (WT‐UeGFP + vehicle: 21366 ± 3320 mean intensity, *n* = 4; prpTDP‐43^A315T^‐UeGFP + vehicle: 7184 ± 1689 mean intensity, *n* = 4; *p* = 0.028; prpTDP‐43^A315T^‐UeGFP + RNS60: 22120 ± 4818 mean intensity, *n* = 4; *p =* 0.032 prpTDP‐43^A315T^‐UeGFP + vehicle versus prpTDP‐43^A315T^‐UeGFP + RNS60; Figure 3A–E).

### 3.5 RNS60 Improves NMJ Integrity

**FIGURE 4:**
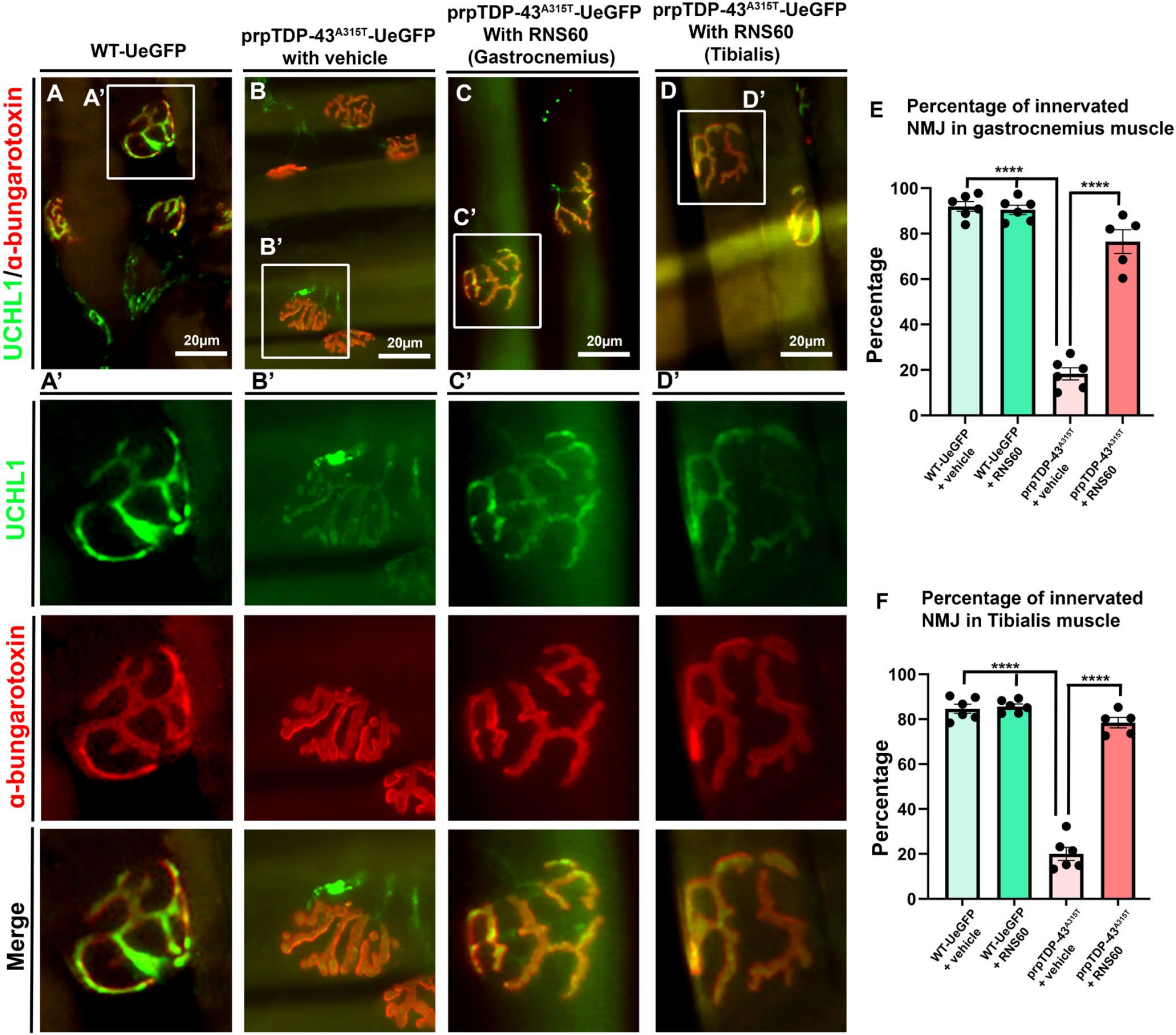
RNS60 preserves the integrity of neuromuscular junctions in leg muscles. (A) Representative images of NMJ from WT‐UeGFP mice treated with vehicle, (B) prpTDP‐43^A315T^‐UeGFP mice treated with vehicle, and (C and D) prpTDP‐43^A315T^‐UeGFP mice treated with RNS60. (E) Quantification of NMJ in gastrocnemius muscle. (F) Quantification of NMJ in tibialis muscle. (A′, B′, C′, and D′) inset magnified and different channels separated for better clarity. ****p = 0.0001. Scale bar: 20 μm.

To assess NMJ integrity, we measured the overlap of staining for the axonal (anti‐UCHL1 antibody) and muscle (α‐bungarotoxin) components of NMJ in gastrocnemius and tibialis muscles, two key limb muscles that display degeneration in ALS. The average percentage of intact NMJ was significantly lower in gastrocnemius muscle of prpTDP‐43^A315T^ mice treated with vehicle compared to WT control mice but significantly increased to levels comparable to WT mice when prpTDP‐43^A315T^ mice were treated with RNS60 (WT‐UeGFP + vehicle: 91.9% ± 2.1%, *n* = 6; WT‐UeGFP + RNS60: 90.5% ± 2%, *n* = 6; prpTDP‐43^A315T^‐UeGFP + vehicle: 18.3% ± 2.6%, *n* = 6; prpTDP‐43^A315T^‐UeGFP + RNS60: 76.5% ± 5.1%, *n* = 5; *p* = 0.0001; Figure 4A–C′,E). Similar results were observed in the tibialis muscle NMJs (WT‐UeGFP + vehicle: 84.6% ± 2%, *n* = 6; WT‐UeGFP + RNS60: 85.6% ± 1.1%, *n* = 6; prpTDP‐43^A315T^‐UeGFP + vehicle: 20% ± 2.9%, *n* = 6, *p* = 0.0001; prpTDP‐43^A315T^‐UeGFP + RNS60: 78.5% ± 2.4%, *n* = 5; *p* < 0.0001, Figure 4D,D′,F).

**FIGURE 5:**
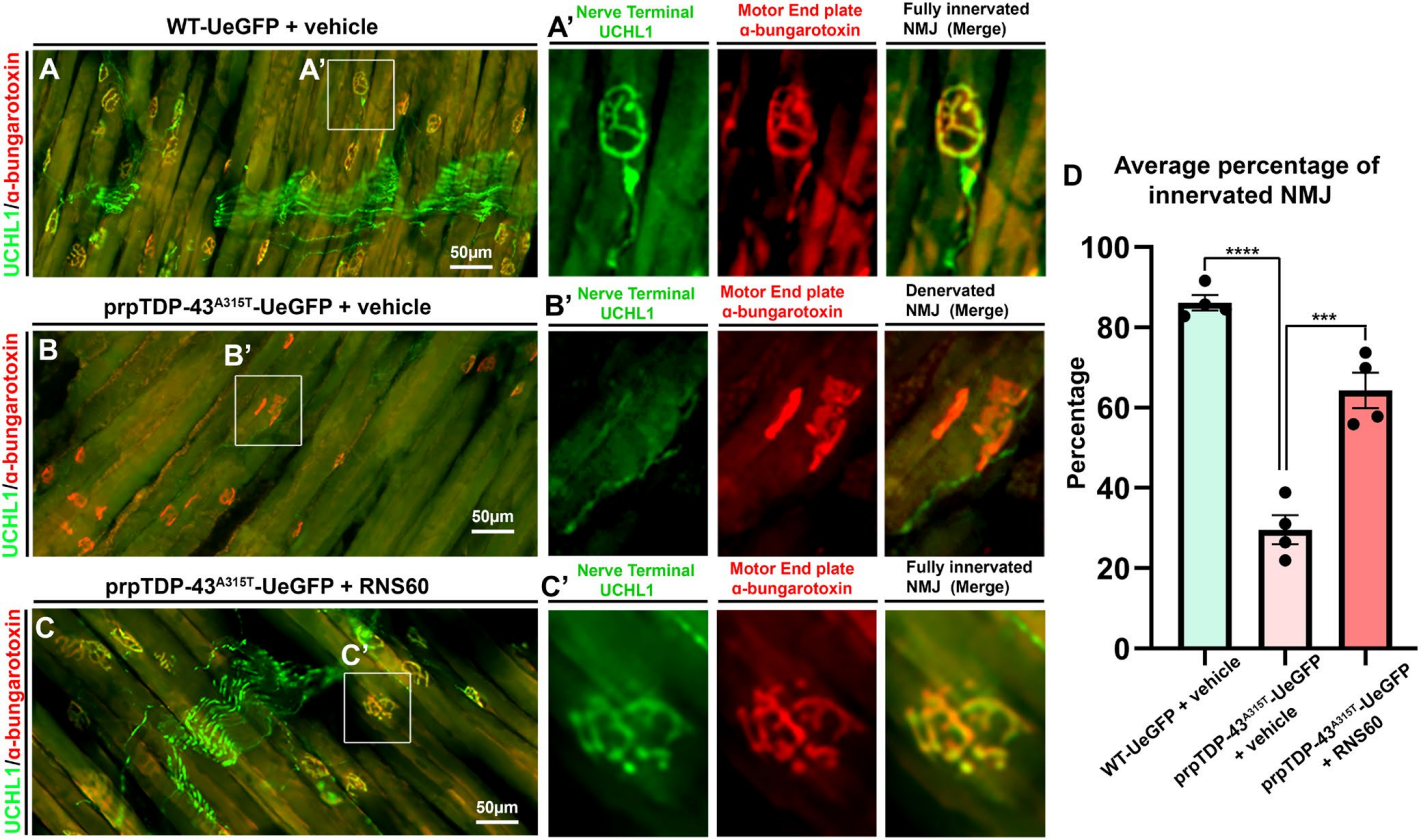
RNS60 preserves the integrity of neuromuscular junctions in the diaphragm. (A) A representative image of NMJ from a WT‐UeGFP mice treated with vehicle. (B) prpTDP‐43^A315T^‐UeGFP mice treated with vehicle, and (C) prpTDP‐43^A315T^‐UeGFP mice treated with RNS60. (D) Quantification of NMJ in diaphragm muscle. (A′, B′, and C′) inset magnified and different channels separated for better clarity. ***p = 0.001, ****p = 0.0001. Scale bar: 50 μm.

We next investigated NMJs in the diaphragm muscle, since RNS60 has previously been shown to slow the decline of breathing function in ALS patients [17]. There was a significantly lower number of intact NMJs in the diaphragm of prpTDP‐43^A315T^ mice compared to WT controls, and RNS60 treatment significantly enhanced the average percentage of intact NMJs in the diaphragm of prpTDP‐43^A315T^‐UeGFP mice when compared to vehicle treated (WT‐UeGFP + vehicle: 86.1% ± 1.9%, *n* = 4; prpTDP‐43^A315T^‐UeGFP + vehicle: 29.6% ± 3.6%, *n* = 4; *p* = 0.0001; prpTDP‐43^A315T^‐UeGFP + RNS60: 64.3% ± 4.4%, *n* = 4; *p =* 0.0002; Figure 5A–D).

## 4 Discussion

Our findings demonstrate that RNS60 treatment improves the structural integrity and function of mitochondria, reduces gliosis, and improves NMJ integrity in three different muscle groups in a TDP‐43 mouse model of ALS. This is hypothesized to result in an overall improvement of motor neuron health and is an important finding as TDP‐43 pathology is found in almost 97% of people with sporadic or familial ALS, where it is associated with mitochondrial defects [4, 5, 29, 30]. Dysregulated TDP‐43 perturbs mitochondrial cristae structure and function in ALS [4, 5, 7]. TDP‐43 directly binds to mitochondrial t‐RNAs and precursor RNA encoded in L‐strand mtDNA resulting in altered mitochondrial function [6] TDP‐43 binds to the mitochondrial protein MFN2 and increasing MFN2 levels were found to reduce TDP‐43 toxicity‐mediated neuronal death [5, 8]. TDP‐43 also triggers mitochondrial membrane pore opening, which ultimately leads to leaking of mtDNA into cytoplasm inciting an immune reaction [9]. Thus, improving mitochondrial health can mitigate TDP‐43 pathology and provide neuroprotection [10, 31].

There is a significant increase of astrogliosis and microgliosis in the motor cortex and spinal cord of both ALS patients and TDP‐43 mouse models of ALS [11, 32]. Gliosis is a hallmark of neurodegeneration. Our studies reveal the ability of RNS60 to reduce gliosis both in the motor cortex and in the spinal cord, which is consistent with and expands on the prior data showing anti‐inflammatory effects of RNS60 in an hSOD1^G93A^ mouse model of ALS [25] and in models of Alzheimer's disease [21] and multiple sclerosis [22].

RNS60 treatment resulted in a favorable outcome with a significant preservation of UMNs comparable to WT numbers and this may be in part because of the multi‐factorial impact RNS60 has on mitochondrial health and gliosis. UMNs integrate cortical input and play a pivotal role in the initiation and modulation of voluntary movement, which is impaired in ALS patients. Recent studies show that UMNs are indeed cellular targets for ALS treatment [33, 34]. Despite their importance and involvement, UMNs have not been sufficiently studied in detail, and there is a need to identify compounds and treatment strategies that preserve this important and disease‐related neuron population. The UMN protection by RNS60 translated into a protection of NMJs of two key limb muscles as well as the diaphragm, muscles that degenerate in ALS [35] patients and in TDP‐43, SOD1, and other mouse models of ALS [36, 37, 38, 39]. Our results show that RNS60 effectively preserved NMJ integrity to levels that were comparable to healthy mice, both in gastrocnemius and tibialis muscles. These findings are also consistent with those of Vallarola et al. in the SOD1^G93A^ mouse model of ALS, where RNS60 treatment reduced NMJ denervation in the tibialis muscle and significantly delayed loss of motor function and paralysis [25].

Respiratory insufficiency is the usual cause of death in individuals with ALS, and breathing is controlled primarily by the diaphragm muscle [40]. In a Phase 2 trial in people with ALS enrolled within 6–24 months of symptom onset, RNS60 treatment over a period of 24 weeks reduced the rate of decline of FVC and this was associated with prolonged long‐term survival [18]. Our finding that RNS60 treatment improved NMJ integrity of the diaphragm muscle after RNS60 treatment may provide a plausible mechanism for the outcomes observed in the clinical trial.

Our goal is to bring cell type specific clarity to the investigation of underlying mechanisms contributing to neurodegeneration so that knowledge with translational power can be generated. This is important especially for complex diseases like ALS, which require comprehensive treatment strategies that address multiple pathways simultaneously. Our findings revealed RNS60's ability to improve multiple disease‐related mechanisms, supporting its further investigation within the context of neurodegeneration and neurorepair.

## Author Contributions

**Danny R. Vesevick:** methodology. **Supurna Ghosh:** writing – review and editing, conceptualization. **Andreas Kalmes:** writing – review and editing, conceptualization. **P. Hande Ozdinler:** conceptualization, writing – original draft, formal analysis, resources, writing – review and editing, funding acquisition. **Mukesh Gautam:** conceptualization, methodology, investigation, writing – original draft, writing – review and editing, formal analysis, funding acquisition.

## Funding

This study is supported by A Long Swim (P.H.O), Queen B Foundation (P.H.O and M.G.) and a grant from Revalesio (M.G.).

## Ethics Statement

We confirm that we have read the journal's position on issues involved in ethical publication and affirm that this report is consistent with those guidelines.

## Conflicts of Interest

S.G. and A.K. are full‐time employees of Revalesio. The other authors declare no conflicts of interest.

## Supporting information


**Figure S1:** Schematic representation of RNS60 treatment strategy.

**Figure S2:** Attenuated gliosis in spinal cord upon RNS60 treatment. (A) A representative image of spinal cord from a WT‐UeGFP mice treated with vehicle. (A') Magnified image of inset. (B) A representative image of spinal cord from a WT‐UeGFP mice treated with RNS60. (B′) Magnified images of inset. (C) A representative image of spinal cord from a prpTDP‐43^A315T^‐UeGFP mice treated with vehicle. (C′) Magnified images of inset. (D) A representative image of spinal cord of a prpTDP‐43^A315T^‐UeGFP mice treated with RNS60. (D′) Magnified images of inset. (E) Quantification of average number of SMN/area. (F) Quantification of average number of activated microglia/area. (G) Quantification of average number of activated astrocytes/area. *p = 0.013, **p = 0.002, ***p = 0.004. Scale Bar: 500 μm (A, B, C, D), 250 μm (A', B′, C′, D′).

**Figure S3:** RNS60 improves mitochondrial ultrastructure in UMN diseased due to TDP‐43 pathology. Representative EM images of Ctip2+ UMN (A) and mitochondria (B, C) from WT mice treated with vehicle. Representative EM images of Ctip2+ UMN (D) and mitochondria (E‐H) from prpTDP‐43^A315T^ mice treated with vehicle. Representative EM images of Ctip2+ UMN (I) and mitochondria (J, K) from prpTDP‐43^A315T^ mice treated with RNS60. Scale Bar: 5 μm (A, D, I), 500 nm (B, F, K), and 200 nm (C, E, G, H, J).

**Figure S4:** SMN Mitochondria ultrastructure is preserved upon RNS60 treatment. Representative EM images of SMN (A) and mitochondria (B, C) from WT mice treated with vehicle. Representative EM images of SMN (D) and mitochondria (E, F) from prpTDP‐43A315T mice treated with vehicle. Representative EM images of SMN (G) and mitochondria (H, I) from prpTDP‐43A315T mice treated with RNS60. Scale Bar: 5 µm (A, D, G), 500 nm (B, C, E, F, H, I).

**Table S1:** Total number of NMJ counted for each treatment group.

## Data Availability

The data that support the findings of this study are available from the corresponding author upon reasonable request.
