# Supplementary figures and images for: Neuroprotective Effects of RNS60 in TDP‐43 Pathology‐Associated Amyotrophic Lateral Sclerosis
Source: Muscle Nerve. 2026 Jun 4;74(2):463–73. doi: 10.1002/mus.70289 (PMC13332579; doi:10.1002/mus.70289)

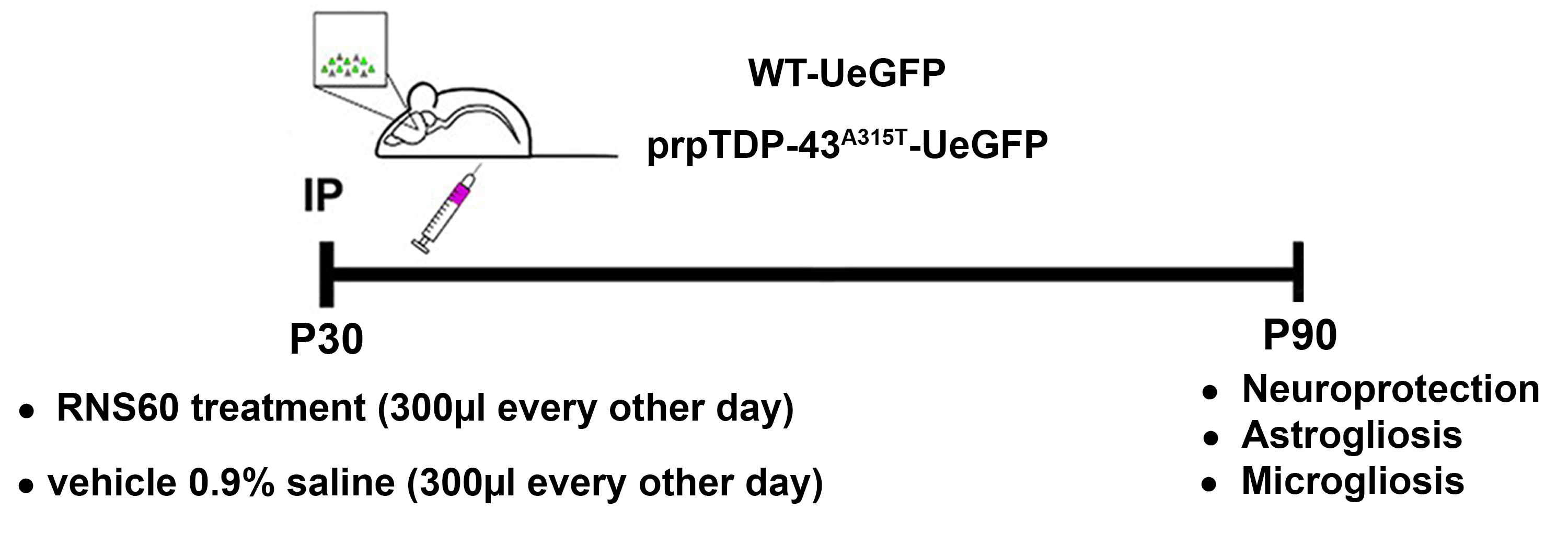

Supplement: Supplementary file 1 — Figure S1: Schematic representation of RNS60 treatment strategy. [file MUS-74-463-s002.jpg]

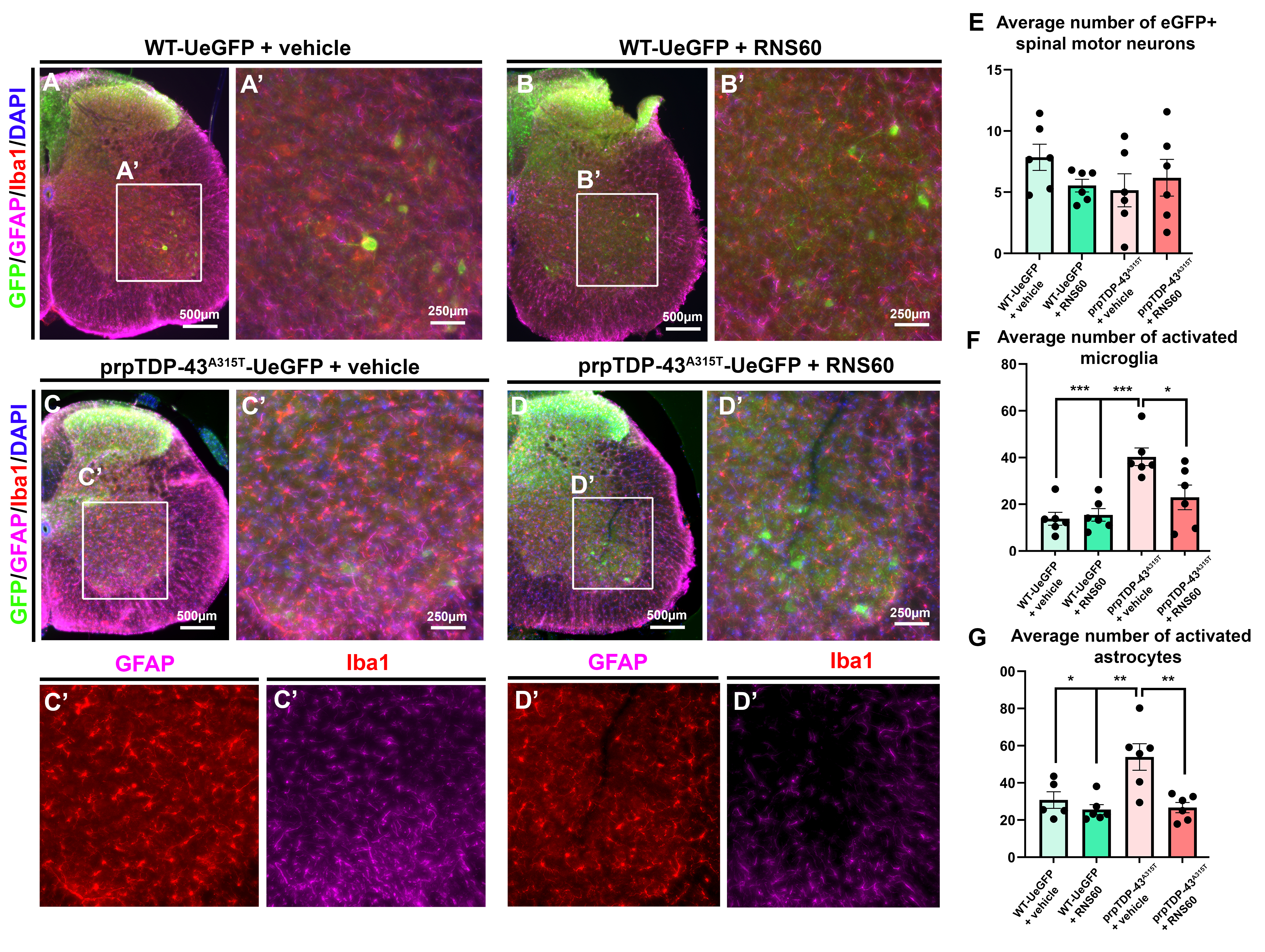

Supplement: Supplementary file 2 — Figure S2: Attenuated gliosis in spinal cord upon RNS60 treatment. (A) A representative image of spinal cord from a WT‐UeGFP mice treated with vehicle. (A') Magnified image of inset. (B) A representative image of spinal cord from a WT‐UeGFP mice treated with RNS60. (B′) Magnified images of inset. (C) A representative image of spinal cord from a prpTDP‐43A315T‐UeGFP mice treated with vehicle. (C′) Magnified images of inset. (D) A representative image of spinal cord of a prpTDP‐43A315T‐UeGFP mice treated with RNS60. (D′) Magnified images of inset. (E) Quantification of average number of SMN/area. (F) Quantification of average number of activated microglia/area. (G) Quantification of average number of activated astrocytes/area. *p = 0.013, **p = 0.002, ***p = 0.004. Scale Bar: 500 μm (A, B, C, D), 250 μm (A', B′, C′, D′). [file MUS-74-463-s005.jpg]

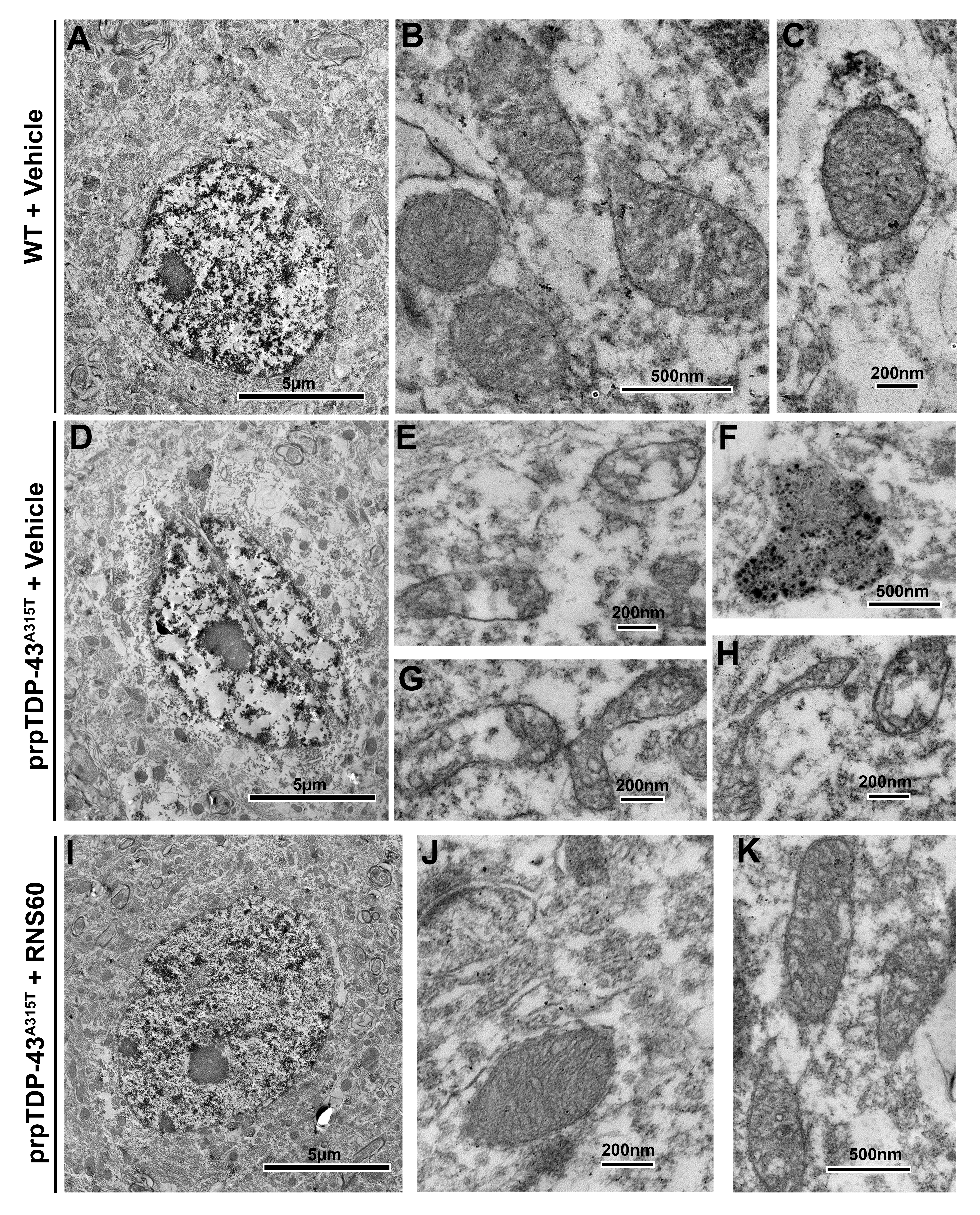

Supplement: Supplementary file 3 — Figure S3: RNS60 improves mitochondrial ultrastructure in UMN diseased due to TDP‐43 pathology. Representative EM images of Ctip2+ UMN (A) and mitochondria (B, C) from WT mice treated with vehicle. Representative EM images of Ctip2+ UMN (D) and mitochondria (E‐H) from prpTDP‐43A315T mice treated with vehicle. Representative EM images of Ctip2+ UMN (I) and mitochondria (J, K) from prpTDP‐43A315T mice treated with RNS60. Scale Bar: 5 μm (A, D, I), 500 nm (B, F, K), and 200 nm (C, E, G, H, J). [file MUS-74-463-s001.jpg]

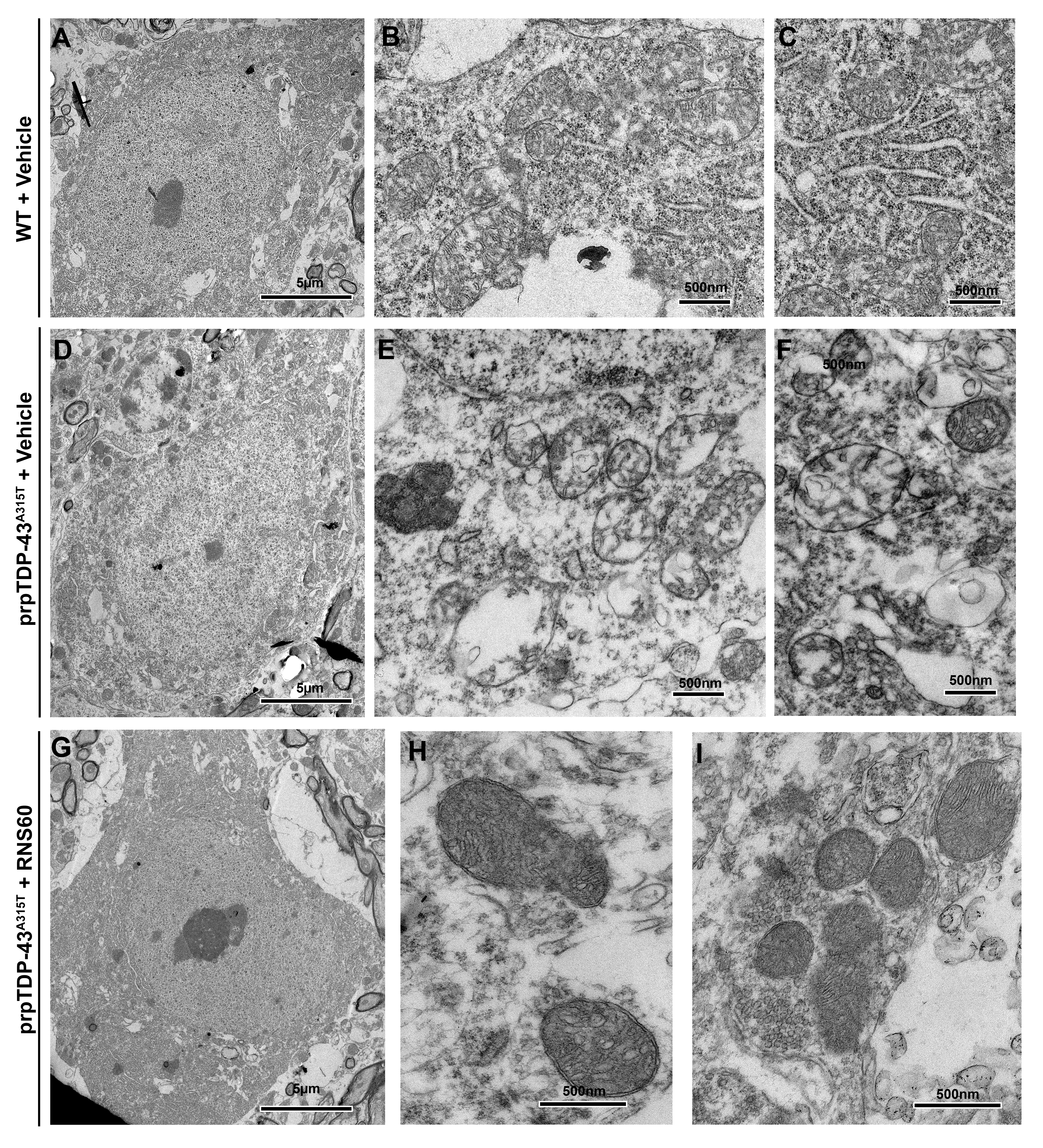

Supplement: Supplementary file 4 — Figure S4: SMN Mitochondria ultrastructure is preserved upon RNS60 treatment. Representative EM images of SMN (A) and mitochondria (B, C) from WT mice treated with vehicle. Representative EM images of SMN (D) and mitochondria (E, F) from prpTDP‐43A315T mice treated with vehicle. Representative EM images of SMN (G) and mitochondria (H, I) from prpTDP‐43A315T mice treated with RNS60. Scale Bar: 5 µm (A, D, G), 500 nm (B, C, E, F, H, I). [file MUS-74-463-s004.jpg]
