# Supplementary material for: Neuroprotective Effects of RNS60 in TDP‐43 Pathology‐Associated Amyotrophic Lateral Sclerosis
Source: Muscle Nerve. 2026 Jun 4;74(2):463–73. doi: 10.1002/mus.70289 (PMC13332579; doi:10.1002/mus.70289)
Supplement: Supplementary file 5 — Table S1: Total number of NMJ counted for each treatment group. [file MUS-74-463-s003.docx]

**Supplementary Table S-1**

Total number of NMJ counted for each treatment group

| WT-eGFP  +vehicle | WT-eGFP  +vehicle | prpTDP43^A315T^UeGFP  +vehicle | prpTDP43^A315T^UeGFP  +RNS60 |
| --- | --- | --- | --- |
| Gastrocnemius Muscle | | | |
| 430 | 386 | 584 | 513 |
| Tibialis Muscle | | | |
| 521 | 512 | 500 | 365 |
| Diaphragm Muscle | | | |
| 846 |  | 1028 | 213 |
